# A Cotton-Fiber-Associated Cyclin-Dependent Kinase A Gene: Characterization and Chromosomal Location

**DOI:** 10.1155/2012/613812

**Published:** 2012-06-14

**Authors:** Weifan Gao, Sukumar Saha, Din-Pow Ma, Yufang Guo, Johnie N. Jenkins, David M. Stelly

**Affiliations:** ^1^Department of Biochemistry, Molecular Biology, Entomology, and Plant Pathology, Mississippi State University, Mississippi State, MS 39762, USA; ^2^USDA/ARS Crop Science Research Laboratory, P.O. Box 5367, Mississippi State, MS 39762, USA; ^3^Department of Plant and Soil Sciences, Mississippi State University, Mississippi State, MS 39762, USA; ^4^Department of Soil and Crop Sciences, Texas A&M University, College Station, TX 77845, USA

## Abstract

A cotton fiber cDNA and its genomic sequences encoding an A-type cyclin-dependent kinase (*GhCDKA*) were cloned and characterized. The encoded GhCDKA protein contains the conserved cyclin-binding, ATP binding, and catalytic domains. Northern blot and RT-PCR analysis revealed that the *GhCDKA* transcript was high in 5–10 DPA fibers, moderate in 15 and 20 DPA fibers and roots, and low in flowers and leaves. GhCDKA protein levels in fibers increased from 5–15 DPA, peaked at 15 DPA, and decreased from 15 t0 20 DPA. The differential expression of *GhCDKA* suggested that the gene might play an important role in fiber development. The *GhCDKA* sequence data was used to develop single nucleotide polymorphism (SNP) markers specific for the *CDKA* gene in cotton. A primer specific to one of the SNPs was used to locate the *CDKA* gene to chromosome 16 by deletion analysis using a series of hypoaneuploid interspecific hybrids.

## 1. Introduction

Cotton fibers are unicellular seed trichomes differentiated from the outer integument of a developing seed. The regulation of cell division is thus an important aspect of fiber initiation and development. About 25% of commercial cotton ovule epidermal cells stops division and develops to produce fibers [1]. It has been reported that the cell cycle in fiber cells is arrested in the G1 phase during the early stages of fiber development [2]. A central role in the regulation of the cell division is played by cyclin-dependent kinases (CDKs) and their regulatory cyclin subunits [3–5]. Eleven types of cyclins (A, B, C, D, H, CycJ18, L, T, U, SDS (solo dancers), and P) have been identified in plants [6, 7]. Plant CDKs, identified in 23 species of algae, gymnosperms, and angiosperms, contain three functional domains: an ATP-binding domain, a cyclin-binding domain, and a catalytic domain. They are classified into five types (A, B, C, D, and E) based on their sequence differences in the cyclin-binding domain [8]. The A-type CDK (CDKA) proteins are characterized by the presence of the PSTAIRE motif, which is essential for cyclin binding [9]. Plant CDKAs, but not CDKBs, have been shown to complement yeast CDK mutants [10–13], suggesting that plant CDKAs are functional homologues of the yeast CDK. Plant CDKAs not only control cell cycle progression from the G1 to S phase and from the G2 to M phase [5, 14] but also participate in cell proliferation and maintenance of cell division competence in differentiated tissues during development [15]. Since the *CDKA* gene is expressed in both dividing and differential tissues [15, 16], it has been suggested that the gene is involved in both cell division and differentiation [17, 18].

 To dissect the possible functional role of CDKA in fiber cell differentiation and development, we have cloned and characterized a fiber *CDKA* cDNA and its corresponding genomic sequences. The expression levels of the *CDKA *transcript and the CDKA protein were also determined in elongating cotton fibers from 5 to 20 DPA ovules and other tissues. The *CDKA* sequence data was then used to develop single nucleotide polymorphism (SNP) markers specific for the *CDKA* gene(s) in cotton. Lastly, a primer specific to one of the SNPs was used with single primer extension technology to locate the *CDKA* gene to chromosome 16 by deletion analysis using a series of hypoaneuploid interspecific hybrids.

## 2. Materials and Methods

### 2.1. Cloning of Fiber *GhCDKA* cDNA

Two degenerate primers (CDK1: 5′-ATHGGDGARGGHACHTAYGG-3′ and CDK2: 5′-CKATCWATCARYARRTTYTG-3′) (H: A + C + T, D: A + G + T, R: A + G, Y: C + T, K: G + T, W: A + T) designed from the conserved ATP-binding and catalytic domains of plant *CDKA* genes were used for PCR to amplify cDNA with homology to the *CDKA* gene using total cDNA from a cotton (*Gossypium hirsutum* L. cv. DES119) fiber cDNA library as template. The cDNA library was constructed using 10 DPA (days post-anthesis) fiber RNA with a Marathon cDNA amplification kit (BD Biosciences, San Jose, CA, USA). A 383 bp DNA fragment was amplified, purified using a QIAEX II gel extraction kit (Qiagen), cloned into pGEM-T Easy Vector (Promega), and sequenced with an ABI PRISM 310 Genetic Analyzer. The DNA sequencing data was analyzed using the BLAST program (NCBI) and LASERGENE software (DNASTAR). Analysis of the sequencing data showed that the 383 bp DNA fragment encoded an A-type CDK. Two gene specific primers CDKC-1 (5′GGCGTTGTTTATAAGGCTCGTGATCGTG-3′) and CDKC-2 (5′CATTCCTTTATCAAATTCTCCGTGGTG-3′) were designed from the 383 bp DNA fragment and used to amplify a full-length *GhCDKA* cDNA by the Rapid Amplification of cDNA Ends (RACE) method with the Marathon cDNA Amplification kit. In the 3′ RACE reaction, CDKC-1 and the adaptor primer AP1 (5′-CCATCCTAATACGACTCACTATAGGGC-3′, 10 *μ*M) were used in the first PCR, and CDKC-2 and the adaptor primer AP2 (5′-ACTCACTATAGGGCTCGAGCGGC-3′) were used in the second (nested) PCR. The 5′ RACEs were also performed as 3′ RACEs, except that primers CDK5-1 (5′-GACACTTTCTCAGGAAGATAGTTG-3′) and CDKC-3 (5′-CCCTATGAGAGTGACAATAAGCAATG-3′) were used in the first and second RACE amplifications, respectively. A full-length *GhCDKA* cDNA was assembled using the 5′ and 3′ RACE products and subsequently confirmed by PCR using *Pfu* DNA polymerase (Stratagene).

### 2.2. Isolation of the Genomic Sequence of the *GhCDKA* Gene

Two primers CDKC-1 and CDK5-1 were used in LA (long and accurate) PCR to amplify DES119 genomic DNA with the Takara LA PCR kit ver.2.1. The PCR was conducted with an initial denaturation at 94°C for 4 min, followed by 30 cycles at 94°C for 30 sec and 68°C for 4 min and a final extension at 68°C for 5 min. A 7547 bp DNA fragment containing the *GhCDKA* gene was amplified. The PCR product was gel purified and cloned, and both DNA strands are sequenced as described above.

The 5′ and 3′ flanking regions of the *GhCDKA* gene were amplified using a PCR-based genomic DNA walking method and inverse PCR. Genomic walking was conducted by amplifying the adaptor-ligated genomic libraries using gene-specific primers GSR-1 (5′-TGAGTTGTGCAGTGAAGTGCATTG-3′) and GSR-2 (5′-CTCTAATTGCAGTGCTAGGTACAC-3′). The self-ligated genomic DNA (previously restricted with *Hin*d III) was used as template in the inverse PCR amplification with primers GSF (5′-TCTGGAAGCGGAAAGAAGCA-3′) and GSR-1 and LA* Taq* DNA polymerase (see Figure  1 in Supplementary Material available online at doi:10.1155/2012/613812.)

### 2.3. Expression Analyses of the *GhCDKA* Gene

Total RNA (10 *μ*g) isolated from various cotton tissues were electrophoresed in a formaldehyde/agarose gel, transferred onto a nylon membrane, and fixed by UV-crosslinking. A 618 bp DNA fragment corresponding to the C-terminal and 3′-UTR region of the *GhCDKA* cDNA was amplified by PCR using two primers CDKC-2 and CDK5-1, labeled with [*α*-^32^P] dCTP with the random priming labeling method, and used as a probe for Northern hybridization. After hybridization, the membrane was stringently washed and exposed to X-ray film for autoradiography. The relative *GhCDKA* transcript levels were determined by the ratio of radioactive intensity of hybridized band of the 1.2 kb *GhCDKA* mRNA to the EtBr stained 28S rRNA using the program of Scion Image for Windows (Scion Corporation). The *GhCDKA* transcript level was also determined by RT-PCR. First strand cDNA, labeled by [*α*-^32^P] dCTP, was synthesized with SuperScript II reverse transcriptase (Invitrogen) using oligo-dT primer and total RNA (2 *μ*g) isolated from flowers, leaves, roots, and 5, 10, and 15 DPA fibers. An equal amount of the synthesized first strand cDNA (based on scintillation counting) from different samples was serially diluted to 1x, 5x, 10x, 20x with sterile distilled water and used as template for PCR amplification with primers CDKC-2 and CDK5-1. Five microliters of the PCR products was analyzed by electrophoresis in a 1% agarose gel.

For Western analysis, 70 *μ*g of total protein extracted from cotton flowers, leaves, and fibers (5, 10, 15, and 20 DPA) with a modified method of Barent and Elthon [19] was vacuum dried and resuspended in SDS-PAGE sample buffer (12 mM Tris-HCl, pH 6.8, 5% (v/v) glycerol, 0.4% (w/v) SDS, 1% (v/v) *β*-mercaptoethanol, 0.02% (w/v) bromophenol blue). The samples were heat denatured, separated by 12% SDS-PAGE, and transferred onto a nitrocellulose membrane. Immunodetection of the GhCDKA protein was carried out with an ECL Western blotting system (GE Healthcare) using rabbit anti-PSTAIRE (Santa Cruz Biotechnology) as primary antibody and anti-rabbit IgG-horseradish peroxidase conjugate (GE Healthcare) as secondary antibody.

### 2.4. SNP Analyses and Chromosomal Location of the *GhCDKA* Gene

Genomic DNAs were extracted from young leaves of CMD-01 (TM-1, *G. hirsutum*), CMD-02 (3–79, *G. barbadense*), CMD11 (*G. tomentosum*), CMD-3 (*G. arboreum*), and CMD-5 (*G. raimondii*) using a DNeasy Plant mini kit (Qiagen). These *Gossypium* genotypes have been widely used for the screening and preliminary characterization of cotton microsatellite markers [20]. The genomic DNA samples were amplified by *pfu* DNA polymerase with two primers (CDKP3, 5′-GGCTGGTTATGTTGTGGTAGTACTG-3′ (nt-913 to -889)); and CDKP4, 5′-GTGCAGCTCCACCAGACGAGAAG-3′ (nt-1 to -23)) designed from 5′-flanking region upstream of the start codon ATG of the *GhCDKA* gene. The amplified PCR products were gel purified, cloned, and then sequenced. The sequence of DES 119 (*G. hirsutum*) was then aligned with those of TM-1 (*G. hirsutum*), 3–79 (*G. barbadense*), and CMD11 (*G. tomentosum*) using the Clustal method (DNASTAR software) for SNP identification.

 The chromosomal location of the *CDKA* gene was determined by following the overall strategy of Liu et al. [21] using hypoaneuploid chromosome substitution stocks (BC_0_F_1_) and a euploid BC_5_F_1_S_1_ chromosome substitution line of TM-1 disomic for the chromosome 16 of *G. barbadense* [22]. The monotelodisomics included telosomes 1Lo, 2Lo, 2sh, 3Lo, 3sh, 4sh, 5Lo, 6Lo, 7Lo, 7sh, 9Lo, 11Lo, 14Lo, 15Lo, 15sh, 16sh, 16Lo, 18Lo, 18sh, 20Lo, 22sh, 25Lo, and 26sh, where Lo = long arm and sh = short arm. Monosomes included chromosomes 1, 2, 3, 4, 6, 7, 9, 10, 12, 17, 18, 20, 23, and 25. Each interspecific hybrid is expectedly heterozygous for all polymorphisms between the two parents, except those rendered hemizygous by the monosome- or telosome-defined deficiency. At hemizygous loci, the *G. hirsutum* allele is expectedly absent and only the *G. barbadense* allele is present. The telosomes expectedly lack all or most of the opposing arm, for example, an F_1_ plant monotelodisomic for 6Lo will be hemizygous for *G. barbadense* polymorphisms in the short arm distal to the telosome breakpoint. We used cytologically identified BC5-derived inbred euploid backcross substitution line for chromosome 16 of *G. barbadense* in *G. hirsutum* in lieu of an available monosomic BC_0_F_1_ plant. The disomic chromosome substitution line is euploid but has one pair of chromosome 16 from *G. barbadense* line 3–79, whereas the other 25 chromosome pairs are largely or completely derived from TM-1.

 A SNP primer (5′-GCCCAACTATAGAAATGAAA-3′) designed based on a single nucleotide differences in the sequences between the lines among the three *Gossypium* species (*G. hirsutum*, *G. barbadense*, and *G. tomentosum*) was used to screen SNP markers of the genetic stocks with the ABI Prism SNaPshot multiplex kit following the method of Buriev et al. [23]. Briefly, the *pfu*-amplified PCR products were incubated with SAP and *Exo* I (5 units of SAP and 2 units of *Exo* I for 15 *μ*L PCR product) at 37°C for 1 hr followed by 75°C for 15 min. The PCR mixture contained 5 *μ*L of SnaPshot Multiplex Ready Reaction Mix, 3 *μ*L of purified PCR product, 1 *μ*L of SNP primer (10 *μ*M), and 1 *μ*L of distilled water. The thermal cycle reaction was carried out with 25 cycles of 96°C, 10 sec, 50°C, 5 sec, and 60°C, 30 sec. After treated with SAP, 0.5 *μ*L of SnaPshot product was mixed with 0.5 *μ*L of size standard and 9 *μ*L of Hi-Di formamide denatured at 95°C for 5 min and then run onto a 3100 Genetic Analyzer (Applied Biosystems).

## 3. Results

### 3.1. Cloning and Characterization of *GhCDKA* Gene

**Figure 1:**
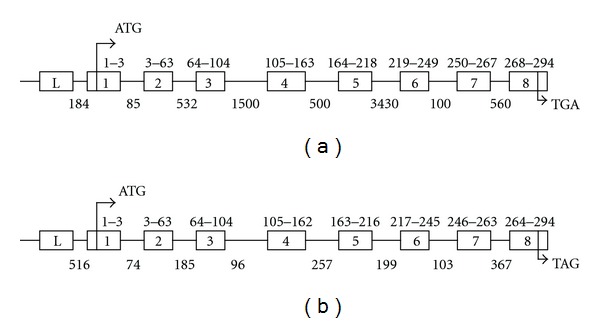
Diagrammatic comparison of cotton *GhCDKA* gene (a) with *Arabidopsis thaliana AtCDKA; 1* Gene ((b), Genbank GI: 18408695). ATG represents the start codon. TGA and TAG are stop codons. The exons containing the coding regions are boxed (numbers 1–8). The exons located in the 5′-UTR region are represented by the L boxes. The positions of codons at the 5′ and 3′ ends within exons are indicated. Intron sizes are indicated under the intron lines.

**Figure 2:**
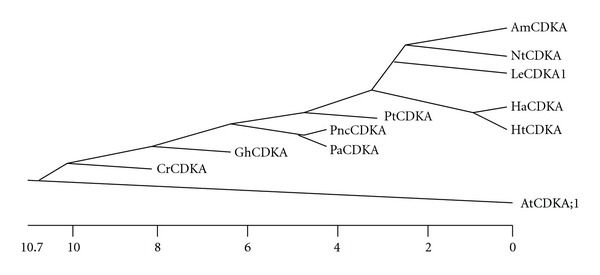
Phylogenetic analysis of eleven plant CDKA proteins. The phylogenetic tree was constructed based on amino acidsequences of 11 plant CDKAs using the Clustal method. The eleven CDKA proteins are GhCDKA (*Gossypium hirsutum*); PtCDKA (*Populus tremula x Populus tremuloides*); HaCDKA (*Helianthus annuus*); PaCDKA (*Picea abies*); LeCDKA1 (*Solanum lycopersicum*); PncCDKA (*Pinus contorta*); CrCDKA (*Chenopodium rubrum*); HtCDKA (*Helianthus tuberosus*); AmCDKA (*Antirrhinum majus*); NtCDKA (*Nicotiana tobacum*); AtCDKA; 1 (*Arabidopsis thaliana*).

A 383 bp DNA fragment was amplified by PCR from a 10 DPA cotton fiber cDNA library using two degenerate primers designed from the conserved ATP-binding and catalytic domains of plant A-type *CDK* genes. BLAST searching in GenBank Databases indicated that the 383 bp cDNA encoded a protein with extensive homology to plant A-type CDKs. A full-length fiber *CDKA* cDNA (1211 bp), named *GhCDKA*, was subsequently cloned by 5′ and 3′ RACEs using gene-specific primers designed from the 383 bp fragment. The *GhCDKA* gene and its 5′ flanking region (9675 bp) (Supplementary Figure 1) were cloned by genomic walking and inverse PCR. *GhCDKA* encodes a protein of 294 aa with a predicted molecular mass of 34 kDa. The protein contained three conserved functional domains of CDK proteins: an ATP-binding domain, a cyclin-binding domain, and a catalytic domain. The GhCDKA protein also had the conserved PSTAIRE motif found in A-type CDKs in the cyclin binding domain. Comparisons of the cDNA and genomic sequences revealed that the *GhCDKA* gene contained 9 exons and 8 introns with 7 introns located within the coding region and one intron at the 5′UTR region (Supplementary Figure 1). The *GhCDKA* gene had the same number and sizes of exons and the same number of introns as the *Arabidopsis CDKA; 1* gene (*AtCDKA; 1*, Genbank GI: 18408695), but the sizes of introns were much larger than those of *Arabidopsis* (Figure 1). The alignment of aa sequences of CDKA proteins from cotton (GhCDKA) and ten other plant species, including *Populus tremula* x *Populus tremuloides* (PtCDKA), *Helianthus annuus* (HaCDKA), *Picea abies* (PaCDKA), *Solanum lycopersicon* (LeCDKA; 1), *Pinus contorta* (PncCDKA), *Chenopodium rubrum* (CrCDKA), *Helianthus tuberosus* (HtCDKA), *Antirrhinum majus* (AmCDKA), *Nicotiana tobacum* (NtCDKA), and *Arabidopsis thaliana* (AtCDKA; 1) revealed that GhCDKA was 91.5–94.2% identical to PtCDKA, PaCDKA, HaCDKA, LeCDKA; 1, CrCDKA, PncCDKA, HtCDKA, AmCDKA, and NtCDK and 86.7% identical to AtCDKA; 1 (data not shown). Phylogenetic analysis of aa sequences of the 11 plant CDKA proteins indicated that GhCDKA was distant to AtCDKA; 1 but closer to the other nine CDKAs (Figure 2).

### 3.2. Expression of the *GhCDKA* Gene

**Figure 3:**
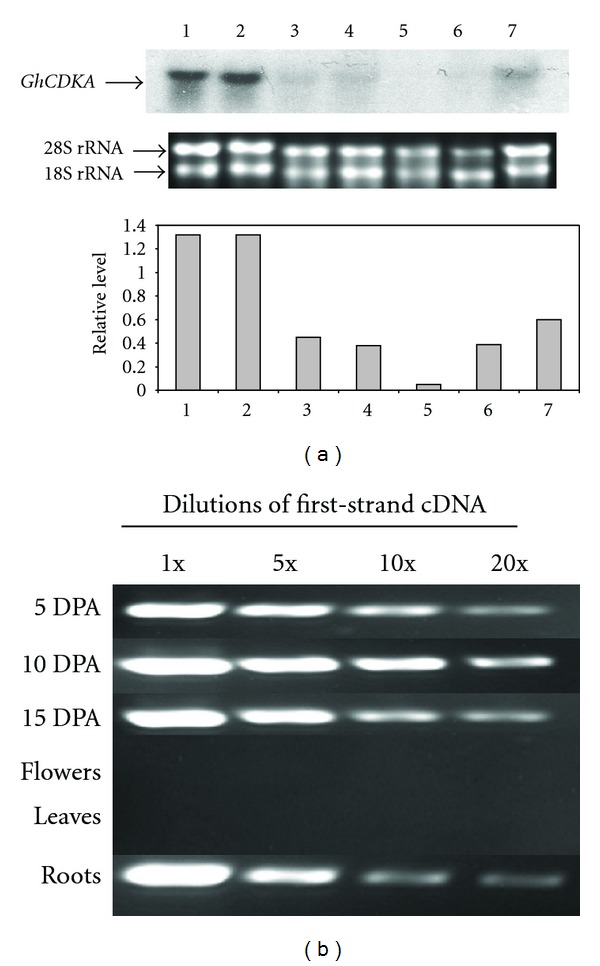
(a) Northern analysis of *GhCDKA* expression in different cotton tissues. Ten *μ*g of total RNA from fibers (5, 10, 15, and 20 DPA, lanes 1–4), flowers (lane 5), leaves (lane 6), and roots (lane 7) was electrophoresed on an agarose gel, transferred onto a nylon membrane, and hybridized with a ^32^P-labeled *GhCDKA* cDNA. Two EtBr-stained rRNA bands indicate that an equal amount of total RNA was loaded for each sample. The relative *GhCDKA* transcript levels were determined by the ratio of hybridized intensity of the 1.2 kb *GhCDKA* mRNA to the EtBr stained 28S rRNA band using the program of Scion Image for Windows (Scion Corporation). (b) RT-PCR analysis of *GhCDKA* mRNA. Total RNA from leaves, flowers, roots, and 5, 10, and 15 DPA fibers was used as template in generating first strand cDNA. Each cDNA was made 1x, 5x, 10x, and 20x dilutions and used as template for PCR amplification with two *GhCDKA* gene specific primers: CDKC-2 and CDK5-1.

The mRNA abundance of the *GhCDKA* gene was analyzed by Northern blot with total RNA isolated from flowers, leaves, roots, and fibers at different developmental stages (5, 10, 15, and 20 DPA). The 618 bp DNA fragment corresponding to the C-terminal and 3′-UTR region of *GhCDKA* cDNA (Supplementary Figure 1) was amplified by PCR with two primers CDKC-2 and CDK5-1 and used as a probe for Northern hybridization. Northern blotting had been performed three times, and the results were similar as shown in Figure 3(a), a 1.2 kb *GhCDKA* mRNA band was detected in all tissues. The *GhCDKA* transcript levels were high in 5 and 10 DPA fibers, moderate in 15 and 20 DPA fibers and roots, and low in flowers and leaves. The *GhCDKA* transcript level was also determined by RT-PCR. As shown in Figure 3(b), the amounts of 618 bp PCR products amplified with the primers CDKC-2 and CDK5-1 were proportional to the first strand cDNA input. The RT-PCR results indicated that transcript levels of the *GhCDKA* gene were high in 5 and 10 DPA fibers, moderate in 15 DPA fibers and roots, and low in flowers and leaves. The RT-PCR result was consistent with Northern analyses.

**Figure 4:**
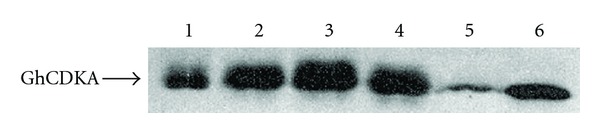
Western blot analysis of GhCDKA. Seventy *μ*g of total protein was subjected to SDS-PAGE, blotted onto a nitrocellulose membrane, and probed with anti-PSTAIRE antibody. Proteins samples are from 5 DPA (lane 1), 10 DPA (lane 2), 15 DPA (lane 3), and 20 DPA (lane 4) fibers, flowers (lane 5), and leaves (lane 6).

Total protein isolated from 5, 10, 15, and 20 DPA cotton fibers, flowers, and leaves was separated by SDS-PAGE, electroblotted onto a nitrocellulose membrane, and probed with anti-PSTAIRE antibody. Western analysis showed that the antibody recognized a 34 kDa protein in all cotton tissues (Figure 4). The GhCDKA protein was present in a moderate level in leaves but low in flowers. The GhCDKA protein in fibers increased from 5 DPA, peaked at 15 DPA, and decreased from 15 to 20 DPA. The Western and Northern results suggest that the *GhCDKA* gene is differentially expressed and developmentally regulated.

### 3.3. Identification of SNP in *GhCDKA*

Analyses of PCR-amplified products from TM-1 (*G. hirsutum*), 3–79 (*G. barbadense*), CMD11 (*G. tomentosum*), CMD-5 (*G. raimondii*), and CMD-12 (*G. mustellinum*) by agarose gel electrophoresis revealed that the products were 0.9 kb in size and not discernibly polymorphic (data not shown). Genomic DNA of CMD-3 (*G. arboreum*) did not yield an amplified product with *CDKA*-specific primers, although this DNA was amplified with other control primers (unpublished information).

**Figure 5:**
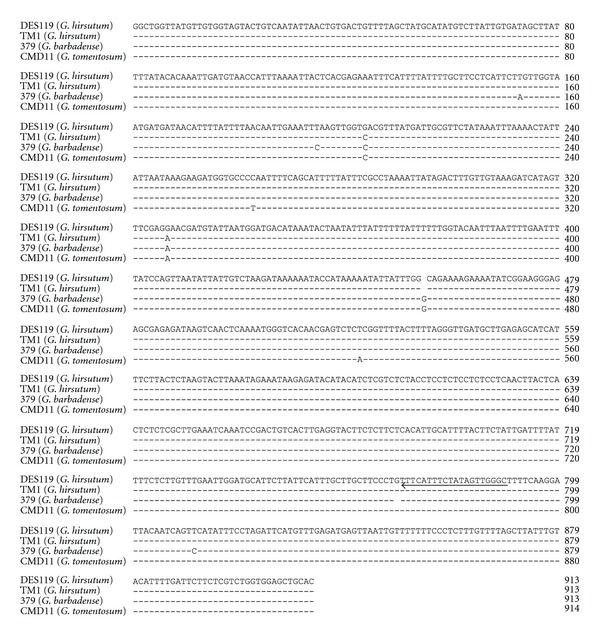
Alignment of 5′ flanking sequences of *CDKA* gene of DES119 (*G. hirsutum*), TM-1 (*G. hirsutum*), 3–79 (*G. barbadense*), and CMD11 (*G. tomentosum*) showing the presence of several SNPs. The arrow indicated the position and the direction of the SNP primer specific to the *CDKA *gene.

 The 0.9 kb 5′ flanking sequence of the *CDKA *gene amplified from genomic DNA of CMD-01 (TM-1, *G. hirsutum*), CMD-02 (3–79, *G. barbadense*), and CMD-11 (*G. tomentosum*), respectively, was aligned with *G. hirsutum* var. DES 119 (Figure 5) for SNP identification. The incidence of SNP was about 1% in the -1 to -913 nt region of the *CDKA* gene. Specifically, we observed two indels, four transversions and three transitions type of mutation in the 5′ flanking sequences of the *CDKA* gene (Figure 5). Two SNP occurred between the two *G. hirsutum* genotypes and six SNP occurred between *G. barbadense* and *G. hirsutum*. Results suggested that a putative *CDKA* locus with at least four different haplotype variants was present in the tetraploid cotton species.

### 3.4. SNP Marker

**Figure 6:**
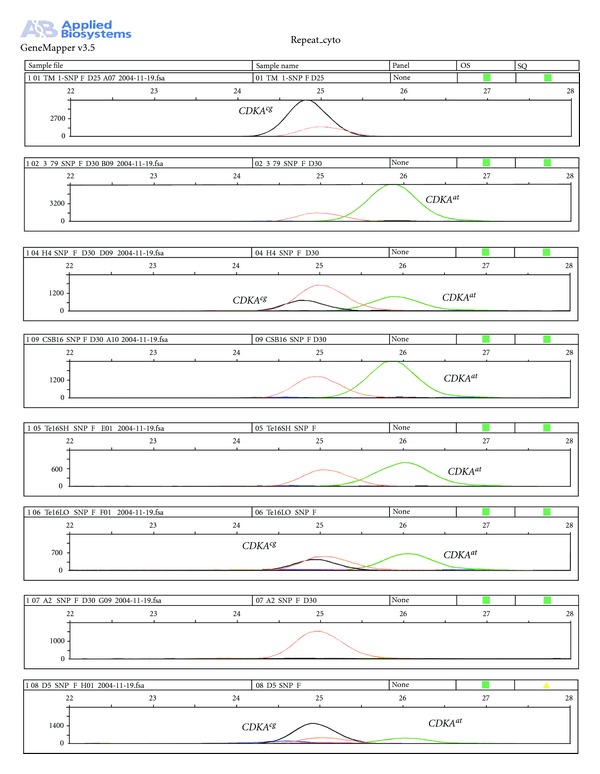
The electropherograms of two allelic SNPs, designated here as *CDKA^cg^* (black) and *CDKA^at^* (green), that corresponded to the polymorphism between *G. hirsutum *inbred TM-1 and *G. barbadense*-doubled haploid 3–79. Genomic dosage profiles are shown for [A] TM-1; [B] 3–79; [C–E] three hypoaneuploid-interspecific *G. hirsutum x G. barbadense* F_1_ hybrids, [C] lacking *G. hirsutum* chromosome 4 (H4), [D] a monotelodisomic-16sh F1 (Te16SH) lacking most of the long arm of *G. hirsutum* chromosome 16, [E] a monotelodisomic 16 Lo F_1_ (Te16LO) lacking most of the short arm of *G. hirsutum* chromosome-16; [F] a backcross disomic substitution plant (CSB-16) in which chromosome 16 of 3–79 has replaced the TM-1 chromosome 16; [G] a *G. arboreum* (A2 species); and [H] a *G. raimondii* plant (D5 species). Electropherograms revealing both peaks that indicate heterozygosity for both parental SNPs, as in the H4 interspecific F1 hybrid, indicate the locus is not in this chromosome. In contrast, absence of the *G. hirsutum *SNP *CDKA^cg^* from CSB 16 and Te16SH hybrids and its presence in Te16LO concordantly indicate that the SNP marker is located in the long arm of the chromosome 16.

To develop a primer for a potentially scorable SNP marker, we targeted a deletion (G) site at nucleotide position 769 (Figure 5), as it distinguished the 3–79 *CDKA* sequence from those of the other tetraploids. The sequence of this specific SNP primer was 5′-GCCCAACTATAGAAATGAAA-3′. Two SNPs corresponding to the TM-1 (*G. hirsutum*) and 3–79 (*G. barbadense*) alleles were identified by the single primer extension technology and designated here as *CDKA^cg^* (black) and *CDKA^at^* (green) (Figure 6). F_1_ hybrids between TM-1 and 3–79 exhibited peaks of both alleles, showing codominance. Our results also detected the presence of *CDKA^cg^* allele in *G. tomentosum* and the presence of both *CDKA^cg^* and *CDKA^at^* alleles in the diploid species of *G. raimondii* (D5). We did not find the presence of any other bases except G or T as SNP markers specific to this SNP primer, suggesting that this locus was biallelic. We did not find the presence of any *CDKA*-specific SNP marker using the genomic DNA of *G. arboreum* (A2) species, suggesting the absence of any such locus in *G. arboreum* specific to the SNP primer or a major change in the primer annealing site of this marker in *G. arboreum*. This result was concordant with the absence of amplified products specific to *CDKA *gene in *G. arboreum* (A2) species, confirming the absence of the *CDKA *gene in *G. arboreum* (A2).

### 3.5. Chromosomal Location

Electropherograms revealed two peaks and thus heterozygosity for *CDKA^at^* and *CDKA^cg^* alleles in all of the hypoaneuploid chromosome substitution F_1_ plants, except one (Figure 6). The single exception was the monotelodisomic Te16sh, which lacks all or most of the long arm of chromosome 16 and possessed the 3–79 allele, *CDKA^at^*, but lacked the TM-1 allele, *CDKA^cg^*. Similar results were observed for the disomic backcrossed chromosome 16 substitution line CSB 16 showing the presence of only the 3–79 alleles.

## 4. Discussion

As a first step toward understanding the mechanisms of fiber cell division and differentiation, a fiber cDNA, *GhCDKA*, and its corresponding gene have been cloned and characterized. The deduced aa sequence of GhCDKA shows high identity (more than 86%) to the CDKAs from 10 diverse plant species. The alignment of the 11 plant CDKAs indicates that they all contain 294 aa residues (except for 302 aa in AmCDKA) and their three functional domains (ATP-binding, cyclin-binding, and catalytic) have identical aa sequences (data not shown). These results indicate that A-type CDKs are highly conserved in higher plants. Comparisons of the cotton *CDKA* gene with the *Arabidopsis cdc2 A* (*CDKA; 1*) gene revealed that both genes contain 7 introns within their ORFs (Figure 1). Although the two *CDKA* genes encode proteins with identical molecular mass, the intron sizes of the two genes are quite different. It will be interesting to examine whether there are any differences in transcriptional regulation or RNA splicing between the two genes. A genome-wide analysis of cell cycle genes indicated that a single *CDKA *gene (*AtCDKA: 1*) exists in *Arabidopsis thaliana* [24]. In contrast, multiple copies of two genes (*LeCDKA1* and *LeCDKA2*) encoding A-type CDKs have been found in tomato [25]. *Nicotiana tabacum* contains a single copy of the *CDKA* gene (*NtCDKA*) and at least one gene similar to *NtCDKA *in the genome [26]. In this study, Southern analysis revealed that one or two copies of the *GhCDKA* gene are present in cotton (*Gossypium hirstum*) (data not shown). *Gossypium hirstum* is a tetraploid plant which contains A and D genomes. Further work is needed to determine whether the *GhCDKA* gene is located in the A or D or both genomes.

The *Arabidopsis* and rice *CDKA* genes have been shown to be expressed not only in dividing tissues of root apex but also in differentiated tissues, such as, sclerenchyma, pericycle, and parenchyma of the vascular cylinder [15, 16]. These results suggest that A-type CDKs are involved not only in cell division but also in cell differentiation which is important to the integration of cell division and differentiation in meristems to produce new organs during plant development. In contrast, no *cdc2* (*CDKA*) transcripts have been detected in differentiated adult tissues of chicken and *Drosophila* [27, 28]. These findings suggest that plant CDKAs may have different functions from those of animals. The *Arabidopsis CDKA; 1* gene (*AtCDKA; 1*) has been shown to participate in trichome morphogenesis and development [29]. Fiber cells grown *in planta* do not divide after initiation; however, some fiber cells can divide under *in vitro* conditions [1]. These observations suggest that fiber cells retain the competence to divide after initiation. In this study, the *GhCDKA* gene has been shown to be strongly expressed in elongated fibers (Figure 3). Western analysis revealed that the fiber GhCDKA protein level increased from 5 DPA, peaked at 15 DPA, and remained at a high level at 20 DPA (Figure 4), which coincided with primary and secondary cell wall syntheses during fiber development. The expression analysis results suggest that GhCDKA may play a role in fiber development. The low *GhCDKA* transcript level versus the high amount of GhCDKA protein in 20 DPA fibers suggests the possible existence of posttranscriptional regulation of the *GhCDKA* gene. In *Arabidopsis*, the transcript and protein levels of AtCDKB; 1 (but not AtCDKA; 1) have been shown to correlate with cell division rate [30].

 Duplications through polyploidization and/or segmental duplication and retrotransposon activity have contributed extensively to the extant genomes of the Malvaceae, including those of *Gossypium* [31–33]. The normal plant cell cycle process is distinguished by a S phase (a round of DNA replication) followed by M phase which are separated by two gap phases (G1 and G2). Previous studies demonstrated that some plant cells followed a different cell cycle mode including endoreduplication where cells undergo iterative DNA replications without any subsequent cytokinesis [34]. Endoreduplication is usually considered to provide a mechanism for increasing cell size [35] and involved modulating the levels of CDKA activity [36, 37]. Cotton fibers are unique cells and they are differentiated from epidermal cells of the ovule. Regulation of cell cycle genes during the very early stages of fiber development triggered some specific epidermal cells in the ovule to stop cell division and then elongate into fiber cells. Previous experiments using 5-aminouracil (5-AU), an inhibitor of DNA replication, demonstrated that cotton fiber cells were arrested at the G1 stage [2]. Our results on Northern blot and RT-PCR analysis revealed that the *GhCDKA* transcript was high in 5–10 DPA fibers and moderate in 15 and 20 DPA fibers. Further studies are needed to reveal if GhCDKA is a regulator of cell cycle and DNA endoreduplication in fiber cells. Duplicated loci pose significant challenges in virtually all aspects of genomics research, including specific gene mapping in tetraploid cotton [23]. Locus-specific markers are thus particularly important for addressing these challenges, and the means to develop them are crucial to the advancement of structural genomics. One possible solution for marker development is to exploit sequence conservation of a specific gene and identify the gene in a locus-specific manner. The *CDK* gene is of special interest because of its possible importance to cotton fiber development, which entails major modifications of cell division and growth. Although cotton is clearly of polyploid origin, agarose gel analyses of amplified PCR product(s) from diverse taxa of cotton genomic DNAs using primers from conserved *CDKA* sequence regions showed no size polymorphisms. Such a result could be due to uniformity across duplicated loci or the existence of just one locus. The predicament had led us to seek SNP markers that could be used to define cotton *CDK* gene(s) and alleles in a locus-specific manner. This approach may be generally applicable for SNP development in cotton and is of particular value for genes that are highly conserved.

 Deficiency tests with interspecific hypoaneuploid F1s provide a quick and fairly robust means of localizing various types of loci to specific chromosomes and arms of cotton. When we examined the hypoaneuploid F_1_ hybrids used here, all but one exhibited a heterozygous banding pattern of *CDKA^at^* and *CDKA^cg^* alleles, suggesting that the *CDKA* locus was in any of respective chromosomes or chromosome arms. However, although *CDKA^at^* was present in the monotelodisomic Te16Lo-interspecific hybrid, it was differentially absent from the quasi-isogenic Te16sh hybrid. These results concordantly localized the *CDKA* gene to the long arm of chromosome 16. In lieu of a monosomic-interspecific F1 hybrid, we examined DNA from a euploid disomic backcross (BC_5_S_n_) substitution line, CS-B16 [38]. The disomic chromosome substitution line is euploid but has one pair of chromosome 16 from *G. barbadense* line 3–79, whereas the other 25 chromosome pairs are largely or completely derived from TM-1. Accordingly, CS-B16 is expectedly devoided of TM-1 chromosome-16 alleles, homozygous for all loci in the alien (3–79) chromosome-16 and also homozygous for TM-1 alleles at nearly all (~99%) other loci of the genome. We observed that only the 3–79 *CDKA^at^* allele is present in CS-B16, strongly supporting the finding from the monotelodisomic interspecific F1 plants that the *CDKA* gene is located on chromosome 16. Our results on the chromosomal location of *CDKA* SNP marker on chromosome 16 were concordant with the cytogenetic evidence on the origin of chromosome 16 from an ancestral D genome diploid species [39].

 The identification of SNP markers enables facile tracking of the *CDKA* gene in cotton, and this gene has been successfully mapped in the long arm of chromosome 16. Our results indicate that single-primer extension technology can be used to identify SNP markers in cotton genes, including the 5′-upstream region of the genes and thus facilitate the mapping and investigation of candidate genes for their effects on fiber development.

## Supplementary Material

The GhCDKA gene and its flanking region (9.7 kb) were cloned by genomic walking and inverse PCR. The gene contains 9 exons and 8 introns with 7 introns located within the coding region and one intron at the 5'-UTR region.

## References

[1] Van’t Hof J, Saha S (1997). Cotton fibers can undergo cell division. *American Journal of Botany*.

[2] Saha S, Van’t Hof J (2005). Cotton fiber cells are arrested at G1 stage. *Journal of New Seeds*.

[3] Dewitte W, Murray JAH (2003). The plant cell cycle. *Annual Review of Plant Biology*.

[4] Inzé D, De Veylder L (2006). Cell cycle regulation in plant development. *Annual Review of Genetics*.

[5] Mironov V, De Veylder L, Van Montagu M, Inzé D (1999). Cyclin-dependent kinases and cell division in plants—the nexus. *Plant Cell*.

[6] Wang G, Kong H, Sun Y (2004). Genome-wide analysis of the cyclin family in arabidopsis and comparative phylogenetic analysis of plant cyclin-like proteins. *Plant Physiology*.

[7] Torres Acosta JA, de Almeida Engler J, Raes J (2004). Molecular characterization of *Arabidopsis* PHO80-like proteins, a novel class of CDKA;1-interacting cyclins. *Cellular and Molecular Life Sciences*.

[8] Joubès J, Chevalier C, Dudits D (2000). CDK-related protein kinases in plants. *Plant Molecular Biology*.

[9] Ducommun B, Brambilla P, Felix MA, Franza BR, Karsenti E, Draetta G (1991). Cdc2 phosphorylation is required for its interaction with cyclin. *EMBO Journal*.

[10] Colasanti J, Tyers M, Sundaresan V (1991). Isolation and characterization of cDNA clones encoding a functional p34cdc2 homologue from *Zea mays*. *Proceedings of the National Academy of Sciences of the United States of America*.

[11] Ferreira PC, Hemerly AS, Villarroel R, Van Montagu M, Inzé D (1991). The *Arabidopsis* functional homolog of the p34cdc2 protein kinase. *Plant Cell*.

[12] Hirayama T, Imajuku Y, Anai T, Matsui M, Oka A (1991). Identification of two cell-cycle-controlling cdc2 gene homologs in *Arabidopsis thaliana*. *Gene*.

[13] Hirt H, Páy A, Bögre L, Meskiene I, Heberle-Bors E (1993). Cdc2MsB, a cognate *cdc2* gene from alfalfa, complements the G1/S but not the G2/M transition of budding yeast cdc28 mutants. *Plant Journal*.

[14] Magyar Z, Mészáros T, Miskolczi P (1997). Cell cycle phase specificity of putative cyclin-dependent kinase variants in synchronized alfalfa cells. *Plant Cell*.

[15] Umeda M, Umeda-Hara C, Yamaguchi M, Hashimoto J, Uchimiya H (1999). Differential expression of genes for cyclin-dependent protein kinases in rice plants. *Plant Physiology*.

[16] Hemerly AS, Ferreira P, De Almeida Engler J, Van Montagu M, Engler G, Inze D (1993). *Cdc2a* expression in *Arabidopsis* is linked with competence for cell division. *Plant Cell*.

[17] Yamaguchi M, Kato H, Yoshida S, Yamamura S, Uchimiya H, Umeda M (2003). Control of *in vitro* organogenesis by cyclin-dependent kinase activities in plants. *Proceedings of the National Academy of Sciences of the United States of America*.

[18] Adachi S, Nobusawa N, Umeda M (2009). Quantitative and cell type-specific transcriptional regulation of A-type cyclin-dependent kinase in *Arabidopsis thaliana*. *Developmental Biology*.

[19] Barent RL, Elthon TE (1992). Two-dimensional gels: an easy method for large quantities of proteins. *Plant Molecular Biology Reporter*.

[20] Yu JZ, Cantrell R, Kohel R Establishment of the standardized cotton microsatellite database (CMD) panel.

[21] Liu S, Saha S, Stelly D, Burr B, Cantrell RG (2000). Chromosomal assignment of microsatellite loci in cotton. *Journal of Heredity*.

[22] Saha S, Wu J, Jenkins JN (2004). Effect of chromosome substitutions from *Gossypium barbadense* L. 3-79 into *G. hirsutum* L. TM-1 on agronomic and fiber traits. *Journal of Cotton Science*.

[23] Buriev ZT, Saha S, Abdurakhmonov IY (2010). Clustering, haplotype diversity and locations of *MIC-3*: a unique root-specific defense-related gene family in Upland cotton (*Gossypium hirsutum* L.). *Theoretical and Applied Genetics*.

[24] Vandepoele K, Raes J, De Veylder L, Rouzé P, Rombauts S, Inzé D (2002). Genome-wide analysis of core cell cycle genes in Arabidopsis. *Plant Cell*.

[25] Joubès J, Phan TH, Just D (1999). Molecular and biochemical characterization of the involvement of cyclin-dependent kinase a during the early development of tomato fruit. *Plant Physiology*.

[26] Setiady YY, Sekine M, Hariguchi N, Kouchi H, Shinmyo A (1996). Molecular cloning and characterization of a cDNA clone that encodes a Cdc2 homolog from *Nicotiana tabacum*. *Plant and Cell Physiology*.

[27] Krek W, Nigg EA (1989). Structure and developmental expression of the chicken CDC2 kinase. *EMBO Journal*.

[28] Lehner CF, O’Farrell PH (1990). Drosophila cdc2 homologs: a functional homolog is coexpressed with a cognate variant. *EMBO Journal*.

[29] Imajuku Y, Ohashi Y, Aoyama T, Goto K, Oka A (2001). An upstream region of the *Arabidopsis thaliana* CDKA;1 (CDC2aAt) gene directs transcription during trichome development. *Plant Molecular Biology*.

[30] Richard C, Granier C, Inzé D, De Veylder L (2001). Analysis of cell division parameters and cell cycle gene expression during the cultivation of *Arabidopsis thaliana* cell suspensions. *Journal of Experimental Botany*.

[31] Moore G (2000). Cereal chromosome structure, evolution, and pairing. *Annual Review Plant Physiology and Plant Molecular Biology*.

[32] Thangavelu M, James AB, Bankier A, Bryan GJ, Dear PH, Waugh R (2003). Happy mapping in plant genome: reconstruction and analysis of a high-resolution physical map of a 1.9 Mbp region of *Arabidopsis thaliana* chromosome 4. *Plant Biotechnology Journal*.

[33] Pfeil BE, Brubaker CL, Craven LA, Crisp MD (2004). Paralogy and orthology in the malvaceae rpb2 gene family: Investigation of gene duplication in Hibiscus. *Molecular Biology and Evolution*.

[34] Leiva-Neto JT, Grafi G, Sabelli PA (2004). A dominant negative mutant of cyclin-dependent kinase A reduces endoreduplication but not cell size or gene expression in maize endosperm. *Plant Cell*.

[35] Sugimoto-Shirasu K, Roberts K (2003). Big it up: endoreduplication and cell-size control in plants. *Current Opinion in Plant Biology*.

[36] Larkins BA, Dilkes BP, Dante RA, Coelho CM, Woo YM, Liu Y (2001). Investigating the hows and whys of DNA endoreduplication. *Journal of Experimental Botany*.

[37] Gonzalez N, Gévaudant F, Hernould M, Chevalier C, Mouras A (2007). The cell cycle-associated protein kinase WEE1 regulates cell size in relation to endoreduplication in developing tomato fruit. *Plant Journal*.

[38] Stelly DM, Saha S, Raska DA, Jenkins JN, McCarty JC, Gutiérrez OA (2005). Registration of 17 upland (*Gossypium hirsutum*) cotton germplasm lines disomic for different *G. barbadense* chromosome or arm substitutions. *Crop Science*.

[39] Endrizzi JE, Turcotte EL, Kohel RJ, Kohel RJ, Lewis CF (1984). Quantitative genetics, cytology and cytogenetics. *Cotton*.

