# Supplementary material for: A Cotton-Fiber-Associated Cyclin-Dependent Kinase A Gene: Characterization and Chromosomal Location
Source: Int J Plant Genomics. 2012 Jun 14;2012:613812. doi: 10.1155/2012/613812 (PMC3382222; doi:10.1155/2012/613812)
Supplement: Supplementary file 1 — The GhCDKA gene and its flanking region (9.7 kb) were cloned by genomic walking and inverse PCR. The gene contains 9 exons and 8 introns with 7 introns located within the coding region and one intron at the 5'-UTR region. [file 613812.f1.doc]

Supplementory Figure 1.

tgaatacggg 10

ttagggtgttatagacattcctagtattatttattagggtgtgacacccactctattcta 70

cttagagtgaaatattgctttctattaaataaatattatttgtactttactagtttaatc 130

atgattctactctctctctttataaatagataaaattggtagaattatttacacttaagt 190

tatacttaagtttgatgtgttgttattttgtcgggaagtagtgataatttatattctaaa 250

aataaattatatttttgaaaattaaaattttaccagtttttattaagagagatttacttt 310

cctactaaaagtaataaatcatttctggttttgtttttggttcatgattgttcgagctta 370

cattcaaagcaattcgtggtaggagaaaaatgaagaaggtagtttggttgaaagccgagg 430

acatctaggatctatctcacacaaaacacaagtatttttcgagaaaaaaaattattacta 490

tacatattacaaatcagctcaattttcaaattttttaatttttcgttggatagaaatctg 550

ttttctaaatcgaatttttccaacacttaaatgttatcttttagtttaagatgaaactga 610

taggagattcctagctaagagtgataaacaatataattagtataagtttatcaacttagt 670

taatgattaacaaattaatcaaccatcgtttttataatttgcatagtcgaaggcaatagg 730

aagaaaatttagttaagtagtttaattgataatttagaattagtctcaatgtacttccat 790

atctggtttgcactaataatttccaacttgattagattagtaattgcttagcttataatt 850

aactcggaaaacacatttaccaaattgacaattctttgagcatattctgagtgttccatt 910

attacacttataaatattataattgacttgaaacacttgtagtaaaagttacttttaatt 970

ctttatatttcctgctaattctttacgttgtgttcatacactaccatgcggtcattatac 1030

accattcgtatggcgcaaatgaaatattttgttaaaaagagagaaatggctggttatgtt 1090

gtggtagtactgtcaatattaactgtgactgttttagctatgcatatgtcttattgtgat 1150

CDKP3

agcttattttatacacaaattgatgtaaccatttaaaattactcacgagaaatttcattt 1210

tattttgcttcctcattcttgttggtaatgatgataacattttattttaacaattgaaat 1270

ttaagttggtgacgtttatgattgcgttctataaatttaaaactattattaataaagaag 1330

atggtgccccaattttcagcatttttatttcgcctaaaattatagactttgttgtaaaga 1390

tcatagtttcgaggaacgatgtattaatggatgacataaatactaatatttattttttat 1450

tttttggtacaatttaattttgaattttatccagttaatattattgtctaagataaaaaa 1510

taccataaaaatattatttggcagaaaagaaaatatcggaagggagagcgagagataagt 1570

caactcaaaatgggtcacaacgagtctctcggttttacttttagggttgatgcttgagag 1630

catcatttcttactctaagtacttaaatagaaataagagatacatacatctcgtctctac 1690

ctcctctcctctcctcAACTTACTCACTCTCTCGCTTGAAATCAAATCCGACTGTCACTT 1750

GAGgtacttctcttctcacattgcattttacttctattgattttattttctcttgtttga 1810

attggatgcattcttattcatttgcttgcttccctgtttcatttctatagttgggctttt 1870

caaggattacaatcagttcatatttcctagattcatgtttgagatgagttaattgttttt 1930

ttccctctttgttttagCTTATTTGTACATTTTGATTCTTCTCGTCTGGTGGAGCTGCAC 1990

CDKP4

ATGGACCAGgtaagtgactcaacccttgtcattccattctgtttctattcacctctagtt 2050

M D Q

ttgctcccttacgctttttttccgaaatttccagTACGAGAAAGTAGAGAAGATTGGTGA 2110

Y E K V E K I G E

GGGAACCTATGGCGTTGTTTATAAGGCTCGTGATCGTGTCACCAATGAAACAATTGCTTT 2170

CDKC-1

G T Y G V V Y K A R D R V T N E T I A L

GAAGAAGATTCGCTTAGAGCAGGAAGACGAGGGTGTACCTAGCACTGCAATTAGAGAAAT 2230

GSR-2

K K I R L E Q E D E G V P S T A I R E I

CTCTCTCTTGAAGGAAATGCAACATGGTAATATCGTCAGgtatgcaatgcacttcactgc 2290

S L L K E M Q H G N I V R GSR-1

acaactcatcctataatcacattgctgtttcaccactcattggtttacactgatttttct 2350

cctgcttgtgctactctttaacgtcggcatatacatttttatcagactacagaactcgtg 2410

ctattgctagttcttcattttgtcttgcctgccaaaggattttaagtttccttttttttt 2470

cattatttaaattttgctcctcctacttttaacataacatggaagtgtggaatcagttat 2530

taaaaaagtttaaggcaacccaaaaaacaaaaagacaataccacacttttctggaagcgg 2590

aaagaagcatatggtattgctctactttctttaagaagtaagtcaacataggcttgttcg 2650

GSF

ttaagcccatgaattttctttttttttatatcaaagttttgaaaccatgatatattctgg 2710

tcatatttcagaatatgagcaaaatccttctcttgtataatgtctgtaatcgtttttccc 2770

tcagcttctctgattgcaccttgggtgacagGTTGCAGGATGTAGTGCACAGTGAGAAGC 2830

L Q D V V H S E K

GTTTATATTTGGTATTTGAATATCTGGACTTGGATTTAAAGAAGCACATGGATTCATATC 2890

R L Y L V F E Y L D L D L K K H M D S Y

CAGAATTTGGGAAAGATCCACGAATGATAAAAgtgagtcagattattcttctgtttgaaa 2950

P E F G K D P R M I K

gtttataagctcaaggtttacctctttggagtttaacagttttaaaatttcacactgcag 3010

tacatgaaattttggtttcatttggctcctttatggaagatggataaattcaaaaaaaga 3070

aaaagaaaaagaaaaagaaaaaagaaaattataatatttataagataagtcaggaacgaa 3130

atgaaataagaattcggcccataaaatctatgttagcttttttgtctgaatgcattctac 3190

atgatccttttggaagagtgggattagaataatacaattttctagtttctttgctctcta 3250

tttctatttgacaaaatccttaggaaaagcaatttgtttccaccaagctaaaacaagatg 3310

taaatgtctctagtaaaccaacgttattgtttaatagctattttgcttctatcccatcaa 3370

aaaaaaggggaagatttagttatgattagaaatacacctttctacctctttatacatgga 3430

tcatttcctcattctattgaaagtattgaactaatgtgaaaaaaaaattgtttgttttgt 3490

aatttcataatccaaatttttaattttaatatataatataattatataattatatataat 3550

ttaattggcccttggatccagatcatgagactgtatatactccctcaaatctttagttga 3610

gaggtaattgtaaaggattaaatccttttaagaaacagatcttttgtttggagtatgaat 3670

caaaccaagggacccttaactcttaaggggattagtagaatgaatcacagttttatgact 3730

aaagtatagtcgctgcaatgcaattattgtatataaaaggaccctttgttgaacagggga 3790

atcgagtgtaatcaagataggattacaagataggagcataaaaaatcctgaaaattaaag 3850

cattgctgcattttgtcagaagactgtgttataggagttttgatagatagtattcaacaa 3910

agggacttagttataacataaaaatgggttctgcagaatccagcgtttttgttatttact 3970

ctagtaaaataaaaggaaataaaaaaaataggaagggaaaaaaagaatagaaattatatt 4030

tgggaagacttaatatagtggttactgttggttttctgcggtttagtagagatgatttac 4090

aaatattttttgaagtgtgacgaattgtggctctttcattggtaatcttttcattgaagt 4150

gtctattagaagccatagagtatgtcatctttgatcttacaccttttatgtgttggacaa 4210

aactaagtagaaatatggtgctgtttgaactgctgttcaagaaatgtctaattgcagaac 4270

ccttctctttatcatattaattaaactattccaggttagcatcttgttccaaatgcataa 4330

atacctagctattatctgtcattggttgaccttggttatctaatggatttggctgatgat 4390

aaatattactcataatggtacttttattatttgatcgtcttgcagGCATTCCTTTATCAA 4450

A F L Y Q

ATTCTCCGTGGCATTGCTTATTGTCACTCTCATAGGGTTCTCCATCGGGATCTAAAACCT 4510

CDKC-2 CDKC-3

I L R G I A Y C H S H R V L H R D L K P

CAAAATTTGCTGATAGATCGCCGTACCAATGCACTAAAGCTTGCTGATTTTGGTCTGGCC 4570

Q N L L I D R R T N A L K L A D F G L A

CGAGCATTTGGTATTCCTGTCAGAACATTTACACATGAGgtactaagaatcaccaagttt 4630

R A F G I P V R T F T H E

gtggttttttgggctgtgcaatgtcatatggtttatgttgctgtgttttgctagacattt 4690

attgtcgattatctctcaaaagtaatgtttcatcttctttctatacaaataataactatt 4750

acaagttggaacaaagaaacttcaattttctggcaaaacgcctggactttgctgcatgtt 4810

tgtaggacaaaagtattttgtttctaattctgacaagaaattctaaggcttaaaatagga 4870

aaagatagtctgacgaactgcaatttgttagtagcatcaaatagtttcctttacatcaat 4930

ttaggattgttgttactgggggtctctaatgcaactttaggcccctatagagcatcagta 4990

taaattagtttttagttggatatgtcctaatgttctagtagagaagtgcttggctataat 5050

ctgcatgctcaatatttcagtcataagcataccaatattggcccggcttctaaagttgtc 5110

gttatgtctagGTCGTTACTTTGTGGTACAGAGCACCTGAAATACTGCTTGGATCTCGCC 5170

V V T L W Y R A P E I L L G S R

ATTACTCTACTCCTGTTGATGTATGGTCAGTGGGCTGTATATTTGCTGAGATGGAGAATC 5230

H Y S T P V D V W S V G C I F A E M E N

AACGGCCATTATTTCCTGGGGATTCTGAGATCGATGAGCTGTTCAAGATCTTCAGgtgtt 5290

Q R P L F P G D S E I D E L F K I F R

gtactctccttattgtagcctccattttattttagtttcaactgtttaagtggtaaagtt 5350

cttacttttgattctcgtgttcttttatctttcttttttgctactgcattcattaaaagt 5410

ctttgctctgataatcttggaatatgttattcaaaaatgatttaggatattatttagtca 5470

tgggtttgtagtgaacatggctgttcttaaatttgcatttcagctaaatttgggttgaat 5530

ttgGatattaagagctaagctgtctcttaaatgttacttgccttgtaaacaagaaaaaaa 5590

aattaacttagctagacAaactcttttagtttttctttggtagaactcttttaaattata 5650

aattggtcgtcttgattatgttctaaattgatagggtcaatatggtttcaggccctaagc 5710

tgttacacttttaacatttggtgcctatagaaaaaaatatcttgatgaggtactctttgt 5770

taataatgtgttagacttggttagctacattagtgtgtttttttcctcaaaaaacatttg 5830

aagttaaaaaaaagatagccggtaaagcgagtcttggaattaggagcatgcttgaatgat 5890

gagtttttatccgattaaacgtgaaatgtagacaatgcaatggaaagaaagcctgccata 5950

gagaagccaaatccttcccatcaaatcaacttgttcattttaatcaatctacttctaata 6010

gatgcatgaaactgaaatatcatacctcataaaactgctgaaatccgtttctctcatctg 6070

gtttcttcttttcttgcacacctaacctcattgtcctatgccaaagctgtctaagagtcc 6130

agcctttaatagttatccacacagttgactcaagaaaccttttgaaaaggtattaggatt 6190

ttgcctagccaagaattataccagtagcttaggctttgtaaaactttcgttagcctttta 6250

tcagttgtctatgtcgtgaacttagaaaaacaaatttttattttttctcaaggagaggtg 6310

cacctaggtttattttttctcaaggaaagcaaactatgtttcacatacacattaagggag 6370

accaaagcaaagcttttatagctaggtttagttgcagtgtgacccttatacttaattgaa 6430

ccattctattatgtgcttgagaagtcattatttctttaatctgagtcctatttcatttta 6490

attcttcaagtcacctttatgtgtgtcccaacatgccaatgaattaaatcaatttggttt 6550

agaattatctatagactgtgaactctagcaatgcatcaaggccaccgaaatgctaggaaa 6610

gtcaatatggtttgacaagtctttcggtgaagagtatcttagtgtaatttgatcccttta 6670

tcaaatatcttggagatggtaagagcatggtgtagaatttactcttaaaccttttcctta 6730

ttacttgagcagcttatatattaatcttttaggtgatgccttggctaaggactttaggtt 6790

aataccggccattagcaagctggattcccatatatcacttaggttgatgagtctttgggc 6850

atcccttgagattgattcatgataaagtcaagcatcacaatccacattgaagtagtgtca 6910

cactagtgtaaattactgaatggaactttcataacaagtttttgggttagtttagcatat 6970

agtggccggcttcttggtttggttccaaaagcacttttaagggtcttttggatggcaaaa 7030

gcccctttttagtgttgcttttactgtcatgtgatctagagtctagccaaccaaaagcaa 7090

tgctaaacacacccatattcattgtacttgttcaacaaactagtggccatatgttatccg 7150

cagtacttttatacttcaacttatgagattggttgacgttcattacatgcatggaagttt 7210

agcatatgctagtctttaagcattgctggtgcctaatctggactatgcaacgactaggaa 7270

cgtttgtagcagcacgactttaatgcataaggatcttatgatctaacttatttacaattc 7330

ctttgcaaggccattactgtttcattggcttatttgcataagttattgctaataagtaat 7390

aacaatatacaccatttatgttaaataaaatataatgtatgtatacatgatattacatgt 7450

ttgtaatagtagaagcaaatgatgagtgatttgtagggcttttacaccaacagagaggcc 7510

aagggagaaggcgagggctaatcaatggcaattatagtttgctcgaactgtccttgcgat 7570

ggtcatccattgccctccccctcctacccacttctctctctcttctcttccttcttgaca 7630

ttcctttatcttcttcttttggaactttcttttttgatttttaaaatgaagtgatttttt 7690

attctagattttttattttttgttaaaaattaaaaataaataccacttcgaacattttta 7750

tttgtgaatattgccacatcagcattttgactaatattgttcgtcagtggcgcaaaatgc 7810

ttgatagaatgtaagttgggtgcctaatcttggataattatttaccttccaaatcttgaa 7870

aggtggtataattcagggtcaaattttgtagtaacctttttttaagccacaattttgctc 7930

ttctgacatttcatactttattcttgacatgtagaagacaatgcgttgtgataagtcata 7990

accatggatacttgtagatacttgtttttggttattaattgttctatcaatagttgtttc 8050

ggggaaatatcgttctttgcatgggcaccttgtcgagaacaaccttggaaggttggcagg 8110

attcttgggttgttccacttttaggaagtcctggaaagtatcgcatggaagctcatcaca 8170

accttcaacgtaactcatttttcttagccacttattgccttcatcaccactatctgccac 8230

ggcacttgttttccagagtctgctccaatctttctctcgaccatctcttcacgacatctc 8290

gttttactagattgtagtgtctcctcaccttaccaaaaaaccctccgcacgtgttttgaa 8350

ttttgctaggctgcaacttgcagctatactgactagaggctaaaagaagagtcatttggc 8410

attagaagatatgaatgacgtcagaaatttttacattgacaactggatttatttcgtatt 8470

attatttttaatttcctcctcaggagtggcctattacggctctttaccggtatttaagag 8530

gtgtatataaccatggatacctttgtacatttatgttcaaagaaaatggagcacttttag 8590

ttttaagtcattgaatataaaagaattgtcttctaggtgtagattgtaggaaaatattgg 8650

tgcaaatttgccccctaaaacttgtgtgttctttttagactgatctagtatcataacatg 8710

tctgtctttttttatgaagAATCTTGGGTACTCCAAATGAGGATACATGGCCTGGAGTGA 8770

I L G T P N E D T W P G V

CTTCATTGCCTGATTTTAAGTCTTCTTTTCCAAAGTGGCCAGCTAAGgtaacatttgtgt 8830

T S L P D F K S S F P K W P A K

ttatggcatctagaggttgtatgttctgttgtgaacctatcagagagacaagtttgtttg 8890

aactaatcaatacaccttttgcttgcagGATTTGGCAACTGTTGTTCCAAATCTTGAATC 8950

D L A T V V P N L E S

TACTGGAATTGACCTTCTTTCTgtaagtatttatttggtgtctttattttttgcttcacc 9010

T G I D L L S

caagtgtgaattacaaagtataagtcagattgatttaatatgaaatatctttcatagttc 9070

ttgagagttgtgagctaaagcgacatgccaaattttttcttatgctaatttgagaaaatt 9130

taattgaaagctttggactattatctcggctctgtttttttttcccgaaataaagagtag 9190

gcttcagacttgagcctagttattacaaaggtttcatgtacctaacactcagtacatcat 9250

taagcaaaagagaccaagcccctggacttgataaattttagtacaatctccattccatct 9310

tcaaagtggctgatcttaataaattttaaaaaagtggcttttcttttgaacttttcttat 9370

ctaaaggctaccatatttctgatatggaacagtttttgaggtctttaacatattattact 9430

attcaggatgatggttttggaataacttcatacacagaaattaagaaccatatatggcat 9490

ctctgcaattttaactgatgatacattctgtctataaatcatccagAAAATGCTGTGCAT 9550

K M L C M

GGATCCCAGCAAAAGAATTACAGCAAGAAGTGCTCTTGAGCATGAATACTTGAAGGATAT 9610

D P S K R I T A R S A L E H E Y L K D I

AGGGTTTGTACCCTGAggataccttttgaaccgctttcccccaactatcttcctgagaaa 9670

G F V P ٭ CDK5-1

gtgtc 9675

Supplementary Figure 1. Nucleotide and Derived Amino Acid Sequences of the *GhCDKA* Gene. Lower case letters represent introns and 5’- and 3’-flanking regions. The upper case letters indicate exons. The derived amino acid sequence is shown under the nucleotide sequence. The ATP binding (GEGTYGVVYK), cyclin binding (EGVPSTAIREISLLKE), and catalytic (HRDLKPQNLLID) domains are underlined. The asterisk indicates the stop codon TGA. The positions and directions of primers CDKC-1, CDKC-2, CDKC-3, CDK5-1, GSF, GSR-1, GSR-2, CDKP3, and CDKP4 are indicated by arrows.
